# Enhanced natural killer cell targeting of cancer stem cells using Cetuximab

**DOI:** 10.1186/2051-1426-2-S3-P13

**Published:** 2014-11-06

**Authors:** Steven K Grossenbacher, Erik Ames, Stephanie Mac, Ragheb Masoud, Robert J Canter, Arta M Monjazeb, William J Murphy

**Affiliations:** 1 Department of Dermatology, University of California, Davis Medical Center, Sacramento, CA, USA; 2 Department of Surgical Oncology, University of California, Davis Medical Center, Sacramento, CA, USA; 3 Department of Radiation Oncology, University of California, Davis Medical Center, Sacramento, CA, USA

## 

Cancer stem cells (CSCs) are a rare population of cancer cells which have been shown to be capable of resisting conventional therapies, such as chemotherapy and radiation. Their persistence following therapeutic intervention, coupled with their stem like nature, endows these cells with the ability to repopulate the tumor, leading to eventual tumor relapse. Previously, it has been shown that the effects of Cetuximab antibody therapy, targeting EGFR on solid tumors, are partially mediated through antibody dependent cellular cytotoxicity (ADCC). Specifically, natural killer (NK) cells possessing the Fc receptor CD16 are important in mediating the anti-tumor effects of EGFR antibodies. Using flow cytometry, and the ALDEFLUOR assay system to identify CSCs, we observed that human pancreatic CSCs express higher levels of EGFR compared to non-CSCs (88.65 vs. 59.7, p < .01) based on median fluorescence intensity. Due to the role of NK cells in facilitating ADCC-mediated anti-tumor responses, and previous work by our laboratory showing that human NK cells can preferentially kill CSCs, we hypothesized that following Cetuximab treatment NK cells could mount a potent cytotoxic response against Cetuximab-bound CSCs. *In vitro *ADCC assays revealed that freshly isolated NK cells from human peripheral blood more effectively killed CSCs in the presence of Cetuximab, as measured using flow cytometry. When NK cells, and either Cetuximab or human IgG, were co-incubated with pancreatic cancer cells at a 5:1 ratio, the presence of Cetuximab resulted in a significantly (p <.001) lower proportion of CSCs remaining after 48 hours, compared to IgG controls (4.22% vs. 14%). Lastly we demonstrate that the combination of local radiation, *ex vivo *IL-2 activated NK cells, and Cetuximab leads to potent anti-tumor effects against subcutaneous human pancreatic tumor xenografts *in vivo*. Primary tumor growth, as measured by external caliper, was significantly delayed in the presence of Cetuximab, compared to untreated animals, up to 28 days following tumor implant (p < .0001). These preclinical results suggest that, alongside conventional cancer therapies such as local radiation, administering Cetuximab in combination with NK cell based therapy could improve clinical strategies to target CSCs and reduce the rate of tumor relapse.

